# Motor Agency: A New and Highly Sensitive Measure to Reveal Agency Disturbances in Early Psychosis

**DOI:** 10.1371/journal.pone.0030449

**Published:** 2012-02-27

**Authors:** Hélène Wilquin, Yvonne Delevoye-Turrell

**Affiliations:** 1 Université Lille Nord de France, Lille, France; 2 UDL3, URECA, Villeneuve d'Ascq, France; 3 Clinique Lautréamont, Loos, France; Royal Holloway, University of London, United Kingdom

## Abstract

**Background:**

Early diagnosis of young adults at risk of schizophrenia is essential for preventive approaches of the illness. Nevertheless, classic screening instruments are difficult to use because of the non-specific nature of the signs at this pre-onset phase of illness. The objective of the present contribution was to propose an innovating test that can probe the more specific symptom of psychosis, i.e., the sense of agency, which is defined as being the immediate experience of oneself as the cause of an action. More specifically, we tested whether motor agency is abnormal in early psychosis.

**Methods:**

Thirty-two young symptomatic patients and their age-matched controls participated in the study. 15 of these patients were at ultra high-risk for developing psychosis (UHR), and 17 patients were suffering from first-episode psychosis (FEP). Patients' neurocognitive capacities were assessed through the use of seven neuropsychological tests. A motor agency task was also introduced to obtain an objective indicator of the degree of sense of agency, by contrasting force levels applied during other and self-produced collisions between a hand-held objet and a pendulum.

**Results:**

As reported in the literature for adult controls, healthy adolescents used more efficient force levels in self than in other-imposed collisions. For both UHR and FEP patients, abnormally high levels of grip force were used for self-produced collisions, leading to an absence of difference between self and other. The normalized results revealed that motor agency differentiated patients from controls with a higher level of sensitivity than the more classic neuropsychological test battery.

**Conclusions:**

This study is in favour of the existence of an abnormal sense of agency early in the psychotic illness. Because it is quick and none verbal, motor agency may be a valuable tool to use in complement to classic interviews, especially when investigating complex ineffable experiences that are difficult to explicitly describe.

## Introduction

Psychosis describes a mental state characterized by distortion or loss of contact with reality and may involve severe disturbances in cognition, behaviour, and emotion. It can be associated to different types of symptoms, one of which may be the positive symptoms that include delusions, hallucinations and thought disorders. Most individuals with schizophrenia, which is the most common of psychosis, experience onset during late adolescence or early adulthood. This initial episode of psychotic disorders can thus be particularly traumatic both for the individuals and their family as it occurs during the key moment in life for the development of identity, relationships and long-term vocational plans. Hence, a particular interest has emerged for the early phase of schizophrenia, including the pre-onset of the illness, i.e., the prodromal period. This is particularly true as more and more evidence show that early clinical intervention, i.e., when the disorder is not yet entrenched, may improve the longer-term outcome of patient [1], [2] and even reduce the duration of untreated psychoses [3].

However, how to provide adequate care to those young adolescences at clinical risk of developing psychosis (Ultra High Risk individuals) remains an unanswered question. This is especially true, as the detection of the emergence of psychosis is rendered difficult by the non-specificity of the prodromal signs and symptoms [4]. The objective of the present contribution was to propose an innovating motor test that might probe the more specific symptom of psychosis, i.e., the sense of agency. This highly sensitive measure could then in a midterm improve the predictive validity of early diagnosis.

Today, the most widely used screening tool for identifying individuals suffering from attenuated psychotic symptoms and thus, at imminent risk for onset of a psychotic disorder is the Comprehensive Assessment of At Risk Mental State (CAARMS) scale [5], [6], [7]. This instrument is based on a “close-in” strategy that combines different risk factors including, e.g., the peak age range for onset of psychotic disorder, the presence of subthreshold psychotic symptoms and signs, family history of psychotic disorders and functional decline [8], [9]. This semi-structured interview provides the means to specify whether an individual meets the Ultra High Risk (UHR) criteria. However, despite the promising identification results obtained with the CAARMS, a recent study investigating the predictive validity of the UHR criteria reported a much lower transition rate (16%) [10] than that reported in the initial cohorts (greater than 40% - for a review see Haroun et al. [11]). More specifically, it was demonstrated that a greater number of “false positive” cases were identified. This may be due to the fact that the questions in the CAARMS do not target the subtle and infraliminary signs that characterise the prodromal phase of psychosis such as basic anomalies in the bodily experiences of self.

Phenomenological studies have argued that the basic sense of self may be one of the earliest and most fundamental features of the abnormal self-disturbance experiences reported in patients suffering from psychosis [12], [13], [14], [15]. This abnormal sense of self would include: (a) a diminished sense of the minimal self, with an inner void and a lack of identity; (b) a distorted first-person perspective, with a pervasive and fluctuating limit between self and the outer-world; (c) an abnormally intensified reflectivity with a circular repetition of thoughts. In UHR individuals, these alterations of the sense of self are rarely of pathological intensity, and it is only during the psychotic transition that they become thematised within the emerging positive symptoms, such as delusions of control and hallucinations. As a consequence, it is difficult to quantify these disturbances at the early stage of the disease, and more particularly through a verbal interview such as the CAARMS, since (1) self- disturbances are so *strange*, that young patients hesitate to express them, and (2) even in the situations for which patients do try to describe their state, the phenomena are so ineffable that they find themselves short of words.

Explicit reports from patients have described an abnormal sense of self as leading to an artificial distance between the body that is moving, and the experience of being. This alienated self-experience would be expressed through thoughts as well as actions leading to abnormal sense of agency. In this way, it has been proposed that one of the most basic components of the sense of self may be the sense of agency, which has recently been defined by Gallagher [16] as being the immediate experience of oneself as the cause of an action.

**Figure 1:**
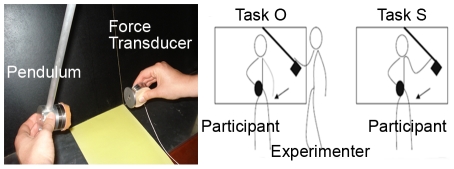
Motor agency task. Illustration of the collision paradigm that was used to estimate the degree of sense of agency experienced by each individual for two different types of events. LEFT: other-triggered collisions with a pendulum that was released by the experimenter; RIGHT: self-produced collisions with a pendulum that was released by the participants. Under both conditions, the subjects were required to remain the hand-held objet immobile and to stop the fall of the pendulum.

Based on the empirical evidence that self-disturbances may be a core feature of schizophrenia and present during the prodromal phase of schizophrenia [15], we have developed the use of a motor agency task [17], [18], [19] to probe the degree of self-disturbances in individuals at clinical risk of developing schizophrenia (UHR individuals). Indeed, we have proposed the use of the collision paradigm [20] because it requires neither reflective responses nor explicit judgments. It provides the means to reveal behaviourally a difference in experiential states between a situation for which the participant is an active agent of the initiation of a collision, and the contrasting situation for which the same participant is passively experiencing collisions that are initiated by someone else. The term “collision” is used because concretely, the subjects' task is to use a hand-held object to resist impacts that are produced as a consequence of the collision between the hand-held object and a pendulum (see Figure 1). The ***degree of agency*** is investigated by contrasting two conditions. In the first, the pendulum is released by the experimenter (other-released, *task O*). In the second, the pendulum is released by the participants themselves (self-released, *task S*). As described in previous studies, a distorted sense of agency is associated to a low-degree of agency, i.e., similar levels of grip force efficiency are applied on the hand-held object when the pendulum is self released (*task S*) and when the pendulum release is triggered by someone else (*task O*). Conversely, a preserved sense of agency is characterised by a significant difference in grip force efficiency between task S and task O.

The originality of this paradigm is to explore the contribution of the pure efferent-based mechanisms by measuring grip force levels at the specific time of impact. Because the duration of the impacts is very short (<30 ms), feedback mechanisms that take a minimum of 90 ms to intervene do not have the time to implement a functional change in grip force adjustments. Thus, we propose that our measure provides the means to probe the participants' immediate experience of being agent or not of that event, without contamination from higher-order cognitive functions mediated through afferent and reflective feedback loops.

Through the use of the collision paradigm, the aim here was first to see whether the sense of agency was indeed perturbed as early as the prodromal phase of psychosis, and to compare these results to those observed in first episode young patients. The second objective was to measure basic motor performances and neurocognitive functions to confirm the none-specificity of general cognitive deficits that are at a sub-pathological intensity. Finally, we aimed at revealing that because it targets the core symptom of psychosis, the motor agency tool is more sensitive than the general neuropsychological battery to detect the emergence of prodromal symptoms.

## Materials and Methods

### Subjects

Thirty-two patients were recruited from a private clinic specialised in the care of adolescents and young adults suffering from mental health diseases, aged between 13 and 25 years of age. Their global functioning was evaluated through the use of the Global Assessment of Functioning Scale (GAF), from the DSM-IV (American Psychiatric Association, 1994). The mean daily dose in chlorpromazine equivalents was also measured. Exclusion criteria were IQ under 70, neurological disorder and drug dependence.

#### First-episode psychosis - FEP Group

Patients, who were admitted to the clinic after a first episode of psychosis, were assessed for diagnoses purposes with the Structured Clinical Interview from the DSM-IV Axis I Disorders. Seventeen patients were included and for each, duration of illness was calculated as the time elapsed since first hospitalisation. Symptom severity was evaluated using the Positive and Negative Syndrome Scale (PANSS [21]). These patients were aged between 16 and 23 years.

#### Ultra High Risk - UHR Group

All patients admitted to the clinic and who did not present a first episode of psychotic illness underwent the semi-structured CAARMS interview, which is specifically designed to monitor pre-pyschotic symptomatology [6]. Fifteen young individuals fulfilled the UHR criteria, with ages ranging from 13 to 23.5 years.

#### Adolescent – ADO Group

Thirty-six healthy young adults aged between 13.5 and 24 years were recruited through local city advertisement, and constituted the control group.

Demographics for the three groups are presented in Table 1. The present study was approved by the local ethics committee: “Comité d'éthique en sciences du comportement de l'Université de Lille 3”. All participants provided written informed consent after the procedure had been fully explained. In the case of subjects under 18 years of age, parental consent was obtained by both parents.

**Table 1:** Demographic characteristics and clinical descriptions of the two clinical groups.

Characteristics	FEP group (N = 17)		UHR group (N = 15)		Between-Patients groups Differences		ADO control group (N = 36)		Between –Group Differences (ADO versus patients)	
*Demographic Characteristics*	*N*	*%*	*N*	*%*	*Kolmogorov-Smirnov(Z)*	*p*	*N*	*%*	*Kruskal-Wallis (X2)*	*p*
Female	8	47	8	53			23	64		

	*Mean*	*SD*	*Mean*	*SD*			*Mean*	*SD*		
Age	20.16	2.62	18.04	2.88	1.218	.036	19.1	3.3	3.171	.205
Education	10.35	2	10.27	2.12	0.255	.999	12.6	2.92	9.399	.009

*Clinical Characteristics*	*Mean*	*SD*				
**Positive and Negative Syndrome scale**						
Positive Component	22.47	7.7				
Negative Component	20.7	6.6				
Excitative Component	6.18	2.9				
Depreciation Component	11.82	4				
Desorganisation Component	16,47	4.98				
**Global Assessment of Functioning Scales Scores**	48.7	15.3	54.83	9.12	1.306	.066
**Duration of illness (weeks)**	83.44	49.9				
**Treatment measures (dose per day at testing)**						
Chlorpromazine equivalents (mg)	261.79	328.25	161.25	161	0.732	.658
Lorazepam equivalents (mg)	3.71	3.71	1.85	0.78	0.745	.635
	*N*	*%*	*N*	*%*		
Untreated	2	12	5	33		
**Drug or Alcohol abuse**	**4**	**24**	**4**	**27**		

### Neurocognitive tests

**Figure 2:**
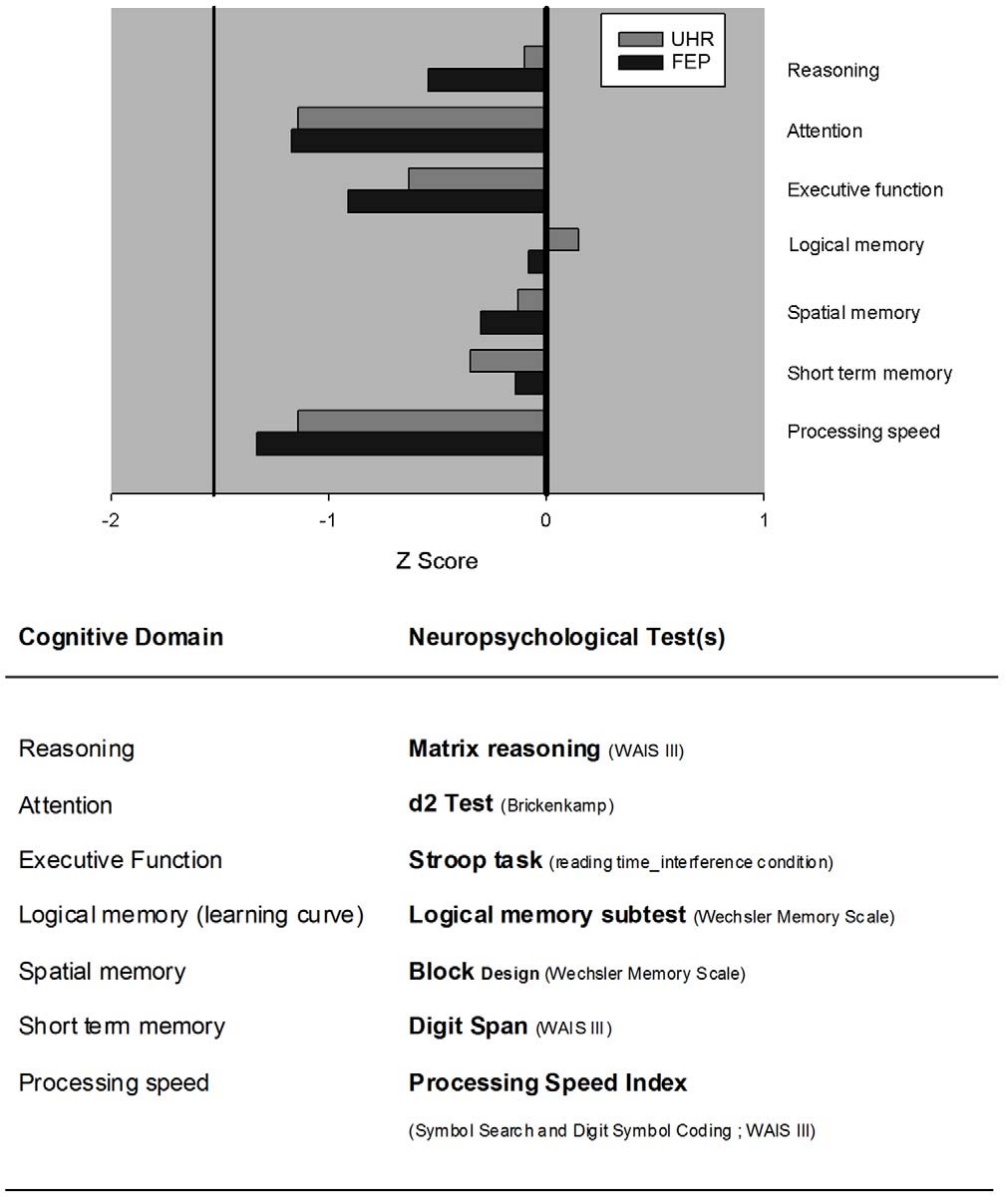
Neurocognitive tests and results. In the neuropsychological test battery, seven neurocognitive domains were assessed for reasoning, attention, executive function, logical memory, spatial memory, short-term memory and processing speed. BOTTOM: for each cognitive domain, the test that was specifically used is defined. TOP: the mean z-scores obtained for the two clinical groups are reported. To note that a value is considered as pathological for a z-score smaller than −1.5 (see Eustache & Faure, 2005).

Seven neuropsychological tests were used to assess neurocognitive function (see Figure 2 BOTTOM). The test battery took 2 hours to complete and was administered in two independent sessions. In addition, the Subjective Scale To Investigate Cognition in Schizophrenia (SSTICS) was proposed [22]. This 21-item self-report questionnaire aimed at exploring those cognitive complaints frequently reported as being impaired in schizophrenia (i.e., memory, attention, executive functions, language and praxia). The total score of the scale only was used. Maximum score was 84, with greater scores revealing higher levels of cognitive complaint.

### Motor agency task

To evaluate the motor and agency capacities of the participants, the collision paradigm was used [20]. The subjects' task was to use a hand-held object to resist impacts produced by the collision between the hand-held object and a pendulum (see Figure 1). Subjects were required to remain immobile and stop the fall of the pendulum that could be released either by the subjects themselves (self-released, *task S*) or by the experimenter (other-released, *task O*). Under all conditions, the hand-held object was fitted with a force transducer (Mini40, *ATI Industrial Automation*) that provided the means to quantify the level of grip force (GF) applied by the subject throughout each trial (in Newtons, N).

The subject's capacity to interact efficiently with the pendulum was evaluated on each and every trial by measuring the **Safety Margin (SM)**, which is an indicator of the excess grip force (GF) employed at the time of impact, with Safety Margin = (Grip Force/Load Force)/Slip Ratio×100%. As suggested by the equation, the smaller the safety margin, the more efficient the interaction [20]. The **objective indicator** of the motor **Sense of Agency (SoA)** was then taken as the **safety margin difference between other-released and self-released collisions** [17]. Subjects were tested within a single 25-minute session and performed a total of 24 trials.

### Statistical method and dependent variables

**Figure 3:**
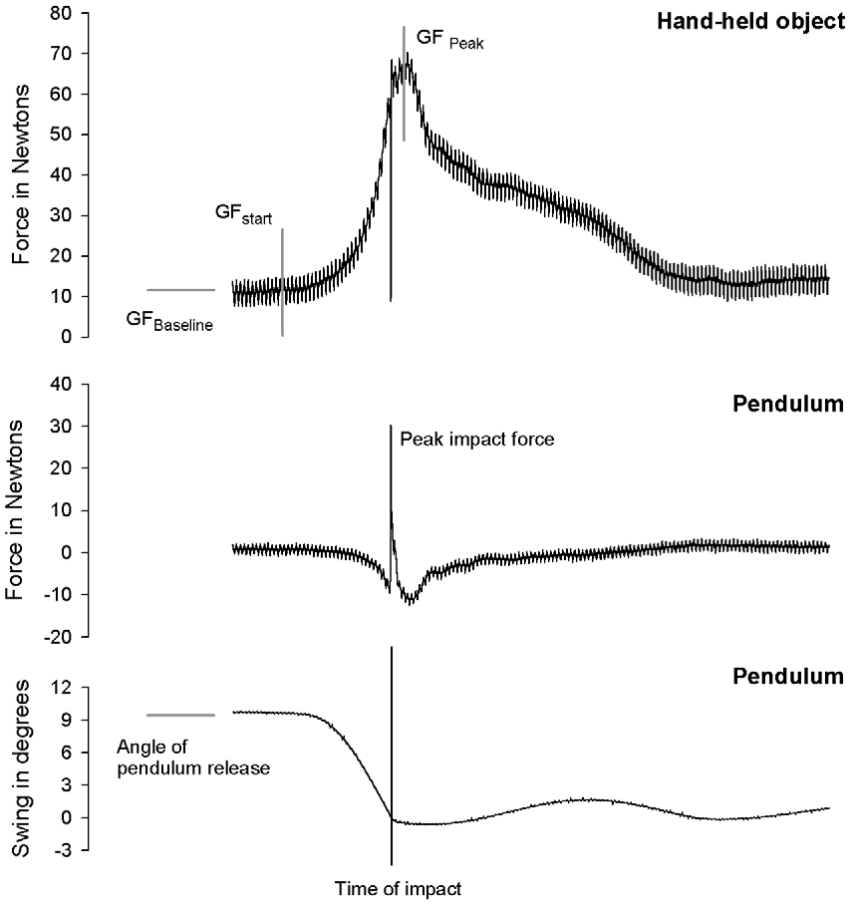
Motor performance measures. This graph presents (top) the grip force (GF) adjustments recorded over the course of a single typical trial for a UHR patient. The two bottom graphs show the impact force applied on the pendulum head at the time of impact, and the variations in angle of pendulum swing, respectively, over the course of the same trial.

In a first series of analyses, nonparametric statistics were used to reveal Group effects (FEP vs. UHR vs. ADO) and Condition effects (Task O vs. Task S). These analyses were conducted with a significance level set at *p* = 0.05. Concerning measures of motor performance (see Figure 3) and agency the following dependent variables were used, extracted from the grip force (GF) measurements:

GF_timing_: time interval between GF peak and time of impact, in milliseconds
GF_planning_: time interval between start of GF increase and impact, in milliseconds
GF_baseline_: force level before pendulum release (first 500 ms of the trial), in Newtons
SoA_indicator_: differences in safety margin (SM) between other and self tasks, in % of excess force

In a second analysis, the sensitivity values of the motor agency parameters and of the neurocognitive tests were calculated as the ratio of (True Positive) over (True Positive+False Negative). True Positive refers to the number of patients identified as having performances that were of pathological level. Conversely, False Negative refers to the number of patients whose performances are not identified as being pathological whereas they should have been. In the present work, we postulated that all patients should present pathological scores even if it was not possible to verify the outcome of the UHR patients.

## Results

### Clinical measures

The clinical groups did not differ with respect to mean age, number of completed years of education, and global functioning scores (GAF). To note, the GAF levels were weak for both clinical groups, and tended to be even lower in the UHR group compared to the FEP group (*P* = 0.066).

At the moment of testing, seven patients were totally untreated (2 FEP; 5 UHR). Overall, 37.5% of the patients were not receiving antipsychotic medication: 17.64% for the FEP group (N = 3); 60% for the UHR group (N = 9). The medicated patients were receiving second-generation antipsychhotics with the exception of one patient. In the FEP group, patients were receiving risperidone (N = 6), aripiprazole (N = 5), olanzapine (N = 1), amisulpride (N = 1) and haloperidol (N = 1). Four patients were also receiving antidepressants. In the UHR group, patients were receiving risperidone (N = 1), aripiprazole (N = 2) and olanzapine (N = 3). Two patients were also receiving antidepressants.

### Neurocognitive evaluations

Raw test scores for both clinical groups were standardized for age using z-score transformations (mean = 0, standard deviation = 1) provided by tests norms. Consequently, the neurocognitive test battery was not run in the ADO group. A z-score below −1.5 was considered as pathological as proposed by Eustache & Faure [23].

Mean results are presented in Figure 2 TOP, and reveal modest neurocognitive deficits in both UHR and FEP groups, across the multiple investigated domains. Furthermore, no significant Group differences were found in any of the seven investigated neurocognitive domains: reasoning (Z = 0.588; *P* = 0.879); attention (Z = 0.514; *P* = 0.954); executive function (Z = 0.556; *P* = 0.916); logical memory (learning curve) (Z = 0.624; *P* = 0.830); spatial memory (Z = 0.588; *P* = 0.879); short term memory (Z = 0.945; *P* = 0.334); processing speed (Z = 0.756; *P* = 0.617).

The results of the SSTICS revealed the presence of important subjective cognitive complaints with no significant between Group differences, with a mean total score of 37.7 (SD = 10.9) in the UHR group and of 37.8 (SD = 11.5) in the FEP group (Z = 0.271; *P* = .711). With a span ranging from 17 to 60, these levels of complaints are even higher than those reported in previous studies of large samples of chronic patients suffering from schizophrenia [22], [24]. These scores confirm the mental stress suffered by the young adults participating in the present study.

### Measures of motor performance and agency

#### Motor control

For the timing aspect of motor control, mean GF_timing_ was calculated and revealed an absence of Group difference with GF peak occurring within 30 ms of impact time for all groups in task S: UHR (32 SD 12 ms), FEP (25.6 SD 19 ms) and ADO (27.2 SD 12 ms) [X^2^(2) = 0.441; *P* = 0.802], and in task O: UHR (32 SD 13 ms), FEP (32 SD 16 ms) and ADO (28.6 SD 14 ms) [X^2^(2) = 4.03; *P* = 0.133]. For the scaling aspect of motor control, GF levels at the time of impact were significantly correlated to the impact force of the forthcoming collision, indicating that all subjects applied greater levels of force when expecting bigger impacts. This was true for all groups (ADO: r_s_ = 0.678; FEP: r_s_ = 0.298; UHR: r_s_ = 0.367) and in both tasks (task S: r_s_ = 0.447; task O: r_s_ = 0.477). For the planning aspect of motor control, mean GF_planning_ was computed and revealed an absence of Group difference with similar time interval between start of GF increase and impact for all groups in task S: UHR (202 SD 55 ms), FEP (227.5 SD 50 ms), and ADO (220 SD 65 ms) [X^2^(2) = 0.329; *P* = 0.84], and in task O: UHR (206 SD 54 ms), FEP (188 SD 29 ms), and ADO (186 SD 27 ms) [X^2^(2) = 0.044; *P* = 0.978]. Overall, these observations indicate preserved motor coordination and motor planning in both clinical groups.

#### Force control

Baseline force measures revealed a main effect of Group [X^2^(2) = 29.72; *P* = 0.001], with greater GF_baseline_ levels in the UHR group (16 SD 9 N) and in the FEP group (9.8 SD 9 N) than in the ADO group (4.5 SD 4 N). This overall lack of efficiency was further confirmed when calculating mean SM, which indicated that an excess of GF was employed at the time of impact in the ADO group (51.8 SD 18%), but that the levels of excess force were significantly greater in the clinical groups [X^2^(2) = 340.485; *P* = 0.001], with the patients in the UHR group (80.7 SD 14%) being even less efficient than those in the FEP group (68.5 SD 19%) – [Z = 3.660; *P* = 0.001].

#### Motor agency

**Figure 4:**
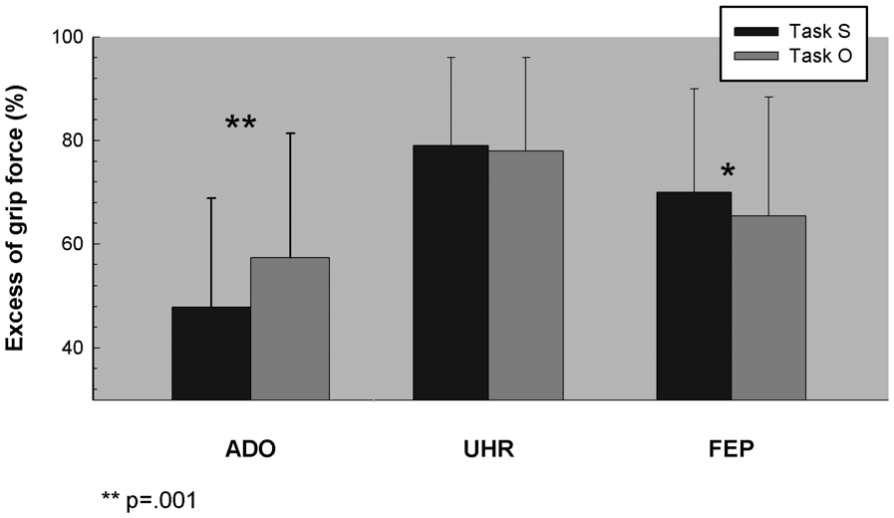
Motor agency perturbation in both clinical groups. Graphic representation of the mean safety margin (SM in %) for the two experimental tasks (O: other-released task; S: self-released task), for the three experimental Groups: adolescent controls (ADO, N = 36); first-episode psychosis (FEP, N = 17); Ultra High Risk (UHR, N = 15).

Motor agency was estimated as the difference in Safety Margin (SM) between other-released (Task O) and self-released (Task S) collisions, SM being an indicator of the excess grip force employed at the time of impact. Results for this parameter are presented in Figure 4. For the ADO group, the Condition effect was significant [Z Wilcoxon = −3.425; *P* = 0.001], revealing greater efficiency in task S (47.9 SD 21%) than in task O (57.4 SD 24%). These results confirm an absence of SoA abnormalities in the ADO group, with the capacity to optimize the motor efficiency of motor adjustments when agent of the collision. These results resemble those reported previously in healthy adults [20]. In the UHR group, the Condition effect between tasks O and S was none significant [Z Wilcoxon = −0.301; *P* = 0.764] suggesting an absence of efficiency difference between task S (79 SD 17%) and task O (80 SD 16%). In the FEP group, the Condition effect between tasks O and S was significant [Z Wilcoxon = −5.482; *P* = 0.001]. However, in contrast to that observed in the ADO group, FEP patients were less efficient in task S (70 SD 20%) than in task O (65 SD 23%).

In order to quantify possible Group×Task interactions, we conducted two non-parametric analyses independently for each Experimental group. Results revealed an absence of Group difference in task O [X^2^(2) = 3.388; *P* = 0.183]. However, Group effect was significant in task S [X^2^(2) = 16.839; *P* = 0.002], with similar results in the two patient groups (*P* = .611). Overall, these results suggest abnormal agency experiences in both clinical groups with a significant alteration of the sense of agency, for self-controlled events specifically.

### Evaluating test sensitivity

The sensitivity values of the 7 neurocognitive tests, on the one hand, and of the parameters of the motor agency task, on the other hand, were calculated after collapsing the two clinical samples together. Results revealed relatively low sensitivity values for the neurocognitive tests: reasoning: 0.18; attention: 0.34; executive function: 0.25; logical memory: 0.09; spatial memory: 0.06; short term memory: 0.13; processing speed: 0.31. For the motor agency parameters, the following results were obtained: GF_baseline_: 0.40; GF_planning_: 0.05; SoA_indicator_: 0.69.

These later results reveal that the parameter of the motor agency is overall more sensitive than the neuropsychological tests classically used. With the greatest sensitivity of all, it is possible that SoA_indicator_ may be more predictive than more general cognitive tests for the early detection of the prodromal phase of psychosis.

## Discussion

The concept of *agency* is related to those phenomenological transactions with the world, in which our basic *sense of self* and the *sense of immersion* in the world are inseparable [25]. In healthy controls, the sense of agency would thus express itself in a tacit and automatic fashion, through interactive activities with the surrounding world. Conversely, an abnormal sense of agency would lead to a basic perturbed sense of self, with an artificial distance between the body that is moving, and the experience of being.

### Objective and results of the study: summary

In the present study, we used a motor agency task to probe the degree of sense of self as being the agent of a collision between a hand-held object and a pendulum. By calculating the efficiency difference between self-initiated and other-imposed collisions, we showed that the sense of agency in healthy young adolescents (13 to 24 years) is similar to that previously evaluated in older adults (greater than 30 years –Delevoye-Turrell et al. [20]). This sense of agency was however abnormal in both young adults suffering from first episode psychosis (FEP) and in those patients detected as being at ultra high risk of developing psychosis (UHR). Our results thus confirm that the sense of agency is perturbed at an early stage of the illness. Such conclusions echo the phenomenological descriptions that have argued that the sense of self is one of the earliest and most fundamental features of the abnormal self-disturbance experiences reported in patients suffering from psychosis [13], [14]. In addition, we demonstrated that motor agency differentiates patients from controls with a higher level of sensitivity than the more classic neuropsychological test battery. As such, it may be valuable to include the motor agency indicator in clinical screening procedures in order to increase the predictive power of the UHR criteria.

### Motor deficits do not explain Motor agency abnormalities

One of the innovating aspects of our work approach was to quantify motor dysfunctions through the use of a force transducer. Indeed, probably due to the limited availability of motor measurement tools, the motor domain is under investigated in the field of psychiatry. In the few studies that have been concerned with movement, (1) retrospective observations were conducted on childhood home movies of schizophrenia patients [26] and (2) prospective observations were run on standardized videotaped footage of the action of eating in offspring of schizophrenic adults [27]. Prospectively studies have also been conducted on unselected birth cohorts [28], [29] and overall, authors report neurological soft signs, poor motor skills and deviances on motor coordination in childhood of to-become patients.

Recently, videotapes were used to investigate the relation between movement abnormalities and prodromal symptoms [30], [31]. In one of their studies, movement abnormalities were coded from videotapes of 40 adolescents at risk of psychosis. Interestingly, comparisons of converted and nonconverted participants at baseline indicated that the groups who converted exhibited significantly more movement abnormalities, suggesting that individuals with elevated movement abnormalities may represent a subgroup of prodromal adolescents who are at the highest risk to develop the illness. Thus, given these findings, we conducted a study in which motor dysfunctions could accurately be described, taken into account both the timing and scaling aspects of movement control.

Motor timing and motor efficiency were evaluated using a hand-held object fitted with a simple force transducer. Results revealed good motor timing, with peak grip force occurring close to the time of impact in both clinical groups. Peak grip force was scaled to the magnitude of the forthcoming collision, confirming that all patients were able to adjust motor response in a predictive matter to the forthcoming impact [32]. Nevertheless, motor efficiency variations revealed that patients used abnormally high force levels at baseline compared to aged-matched controls, confirming certain aspects of the neurological soft signs previously reported. These findings are also consistent with results demonstrating that self-generated forces are less attenuated in schizophrenia patients in comparison with healthy subjects, thereby enhancing higher grip force levels [33]. Importantly, in the present study motor dysfunctions where similar in all points, under both collision tasks (Self and Other).

### Motor agency is impaired in early psychosis

The critical aspect of our protocol is the contrast between the task of arresting the fall of a pendulum that was initiated by the experimenter (Task Other) or that was initiated by the subject (Task Self). The motor efficiency differences between self and other, measured at the time of impact was then taken as the indicator of his/her immediate experience as an agent of that collision. Hence, an absence of efficiency differences between these two conditions (Self and Other) will reveal an altered sense of agency.

Results revealed that healthy adolescents were significantly more efficient when the pendulum release was self-initiated than when it was released by the experimenter suggesting that healthy individuals pre-reflectively experience self-initiated events as different from externally initiated ones. These findings confirm those previously reported in older adults reporting best efficiency for the self-controlled situations whether acting in impoverished environment or not [20].

The motor agency indicator revealed however abnormal results in both of the patient groups. More specifically, force levels in the self-controlled task were abnormally scaled. On the one hand, UHR patients set similarly force levels for task-S and task-O, suggesting that they experienced both types of collisions as similar, whether they were agent of pendulum release or not. On the other hand, FEP patients were even less efficient in Task-S than in Task-O, with an inverse pattern of results than the age-matched controls. These findings are similar to those described in chronic schizophrenia [17], [34], suggesting an early deficit in the sense of agency in psychotic illnesses. Our results also agree with findings of a recent study, using time judgment of action, which reported distortions of sense of agency in putative psychotic prodrome [35], [36].

### Neuropsychological evaluation is less sensitive than motor agency

Through the use of a neurocognitive tests battery, we obtained a precise clinical description of the patients who participated in the present study. In doing so, we confirmed relatively poor neuropsychological abilities in both the UHR and the FEP patients with scores for sustained attention, executive function and processing speed that were close to being pathological. The results presented here resemble previous findings for first episode psychosis [36] as well as for UHR individuals and young adults at genetic risk [37], [38], [39], [40]. To note is the fact that the levels of global functioning in our UHR group were as weak as that reported by Brewer and collaborators [37] for those UHR patients who truly did develop full-blown psychosis. This argues in favour for the fact that the individuals in our UHR group, truly are in a putative prodromal psychotic phase, even if it will remain unknown how many of these individuals will truly go on to develop psychosis.

Finally, statistical analyses provided us the means to reveal low sensibility values for the cognitive tests that are classically used in clinical interviews (values ranging from 0.09, for memory to 0.34, for attention). These values were all lower than those found for the motor parameters. Interestingly, the sense of agency indicator was the one that revealed to be the most sensitive to distinguish pathological from none pathological individuals, suggesting that the motor agency task may be a valuable tool to use in complement to classic screening interviews in order to help detect individuals at risk of psychosis.

### Conclusions

We have reported the importance of considering quantifying methods as complementary to classic questionnaires, especially when investigating subjective experiences that are difficult to verbalise. The motor agency indicator is a simple non-verbal test that provides within 20 minutes an objective indication of the degree of alterations of the sense of agency, one of the most fundamental features of the schizophrenic spectrum [41]. With a high degree of sensitivity, motor agency could be an important parameter to include in clinical screening to increase the predictive power of the UHR criteria for early diagnosis.
